# Assessment of Knowledge and Attitudes Related to Breath-Holding Spells Among Adults in Riyadh, Saudi Arabia

**DOI:** 10.7759/cureus.110373

**Published:** 2026-06-06

**Authors:** Rimah O Alfawzan, Saba A Algashami, Rana I Alrgaiba, Mohammed Almesned

**Affiliations:** 1 Family Medicine, King Saud Medical City, Riyadh, SAU

**Keywords:** attitudes, breath-holding spells, knowledge, primary health care, saudi arabia

## Abstract

Background: Breath-holding spells (BHS) are benign, self-limiting episodes in children that often cause parental anxiety. Adult awareness is crucial to mitigate undue panic and prevent inappropriate medical interventions, yet public understanding of BHS is limited, particularly in Saudi Arabia.

Aim: To evaluate and quantify the levels of knowledge, awareness, and attitudes toward BHS among adults in Riyadh, and to identify specific socio-demographic factors significantly associated with good knowledge scores using a structured cross-sectional design.

Methods: A descriptive cross-sectional study was conducted among 444 Saudi adults in Riyadh, Saudi Arabia. Data were collected using an expert-reviewed, pilot-tested online questionnaire. Knowledge scores were calculated based on correct responses (≥60%=good knowledge). Data were analyzed using IBM SPSS Statistics for Windows, Version 28 (Released 2021; IBM Corp., Armonk, New York, United States), and associations were tested using the chi-square test.

Results: Most participants were female (n=367, 82.7%) and married (n=287, 64.6%), with a bachelor’s degree (n=258, 58.1%). Only 44.4% (n=197) had heard of BHS, and 24.3% (n=108) demonstrated good knowledge. The main sources of information perceived as helpful were the internet (n=207, 46.6%), family/friends (n=195, 43.9%), and healthcare providers (n=192, 43.2%), although only 9.5% (n=42) had actually received direct BHS information from a provider previously. Younger adults (18-25 years) and single participants had significantly higher knowledge (p<0.05), while gender, education, income, and parental status showed no significant association. Most participants reported high anxiety regarding episodes (n=339, 76.4%) and indicated they would seek medical advice for recurrent spells (n=278, 62.6%) regardless of knowledge level.

Conclusions: Knowledge and awareness of BHS in Riyadh are suboptimal despite high concern and willingness to seek care. Age and marital status were the only significant predictors of knowledge. These findings highlight the need for primary care-based education to improve public understanding of BHS, reduce anxiety, and minimize unnecessary healthcare utilization.

## Introduction

Breath-holding spells (BHS) are known as common, benign, non-epileptic paroxysmal events predominantly observed in infants and young children, typically manifesting between 6 and 48 months of age [1]. These episodes are frequently precipitated by emotional stimuli, such as anger or frustration, or minor trauma [2]. BHS are categorized into three primary types based on observed changes in skin coloration: cyanotic, pallid, and mixed [2,3]. While pallid BHS is generally attributed to an excessive vagally mediated cardiac inhibition, the underlying pathophysiology of the more prevalent cyanotic type remains more complex and not yet fully explained [4].

Globally, BHS represents a significant, though often misunderstood, burden within pediatric populations. Although BHS are largely considered benign and do not typically lead to severe neurological damage, recurrent or particularly severe episodes have the potential to induce transient cerebral hypoxia, which can understandably cause considerable distress for parents and caregivers [5]. In very rare and extreme scenarios, children experiencing severe BHS might necessitate medical interventions such as pacemaker implantation [6,7]. Clinical presentations of BHS commonly involve a period of apnea and alterations in postural tone that follow a crying episode [2,8]. Emerging research also suggests a potential association between BHS and sleep disturbances in affected children [2,9]. Furthermore, a strong familial predisposition for BHS has been documented, with case reports detailing instances where multiple siblings within a single family experienced these spells, sometimes with onset as early as the neonatal period [1,10]. While the incidence of BHS typically peaks around two years of age and spontaneously resolves by five years, exceptional cases have been reported, such as cyanotic spells occurring in neonates as early as three days postpartum [11].

The global prevalence of BHS is estimated to range between 0.1% and 4.6% in healthy children, making it a relatively common phenomenon that healthcare providers and parents encounter [12]. This prevalence contributes to the burden by frequently prompting parental concern and medical consultations, even when the condition is benign. In Saudi Arabia, while specific national epidemiological data are scarce, the clinical impact of BHS on parental anxiety and pediatric consultations directly reflects the established global burden [12].

Given the prevalence of BHS and the potential anxiety it can induce in families, understanding the knowledge and context status of the Saudi public regarding this pediatric condition holds substantial public health importance. Such research can help to identify common misunderstandings, such as misdiagnosing BHS as epileptic seizures or showing either excessive worry or neglect concerning the severity of the spells [2,8]. By evaluating these aspects within the Saudi Arabian context, targeted health education programs can be developed to improve accurate public understanding of BHS, lessen unnecessary parental anxiety, and ensure that affected children receive appropriate attention and management, thereby reducing the burden of disease misinformation and promoting better child health outcomes.

Therefore, this study aimed to assess the knowledge, awareness, and attitudes toward BHS among Saudi adults in Riyadh, while identifying the socio-demographic factors associated with these levels.

## Materials and methods

A descriptive cross-sectional study utilizing a non-probability convenience sampling technique was conducted among Saudi adults aged ≥18 years in Riyadh. Healthcare professionals, individuals <18 years, and incomplete questionnaires were excluded. Assuming a large target population size of adults in Riyadh, the minimum sample size was calculated using Cochran’s formula. An expected prevalence of 50% was selected to maximize variance and ensure a conservative sample size estimate. With a 95% confidence level and a 5% margin of error, the formula yielded a requirement of 385 participants. A final sample of 444 fully completed and valid questionnaires was achieved and included in the analysis after excluding all incomplete responses.

Data were collected via a self-administered online questionnaire consisting of 19 items distributed across three sections (see Appendices). The questionnaire link was disseminated electronically through popular social media platforms and instant messaging applications (such as WhatsApp and Twitter/X) to reach the target population in Riyadh. The questionnaire was initially developed in English, forward-translated into Arabic to match the general public in Riyadh, and back-translated to maintain conceptual consistency. Face and content validity were verified by a panel of expert consultants. Because the tool utilized nominal, categorical, and multi-select questions rather than a continuous psychometric scale, a standard Cronbach’s alpha was not computed; instead, the questionnaire was pilot-tested with 20 adults to ensure clarity, readability, and structural reliability before final distribution, and their data were completely excluded from the final statistical analysis.

The study adhered to the Declaration of Helsinki. Ethical approval was officially obtained from the Institutional Review Board at King Saud Medical City, Riyadh, Saudi Arabia (approval no.: H1RI-01-May25-04) on May 4, 2025. Participation was voluntary, informed consent was obtained electronically from all participants prior to data collection, and all data were kept confidential and anonymous.

Data were analyzed using IBM SPSS Statistics for Windows, Version 28 (Released 2021; IBM Corp., Armonk, New York, United States). Descriptive statistics summarized demographics, knowledge, attitudes, and information sources. Associations between knowledge and demographic/attitudinal factors were assessed using Pearson chi-square tests, with exact probability tests applied when expected counts were <5. A p-value <0.05 was considered statistically significant.

The knowledge section comprised seven items, with a total possible score ranging from 0 to 7. Scoring was defined by assigning one point for each correct question and 0 points for incorrect or "don't know" responses. For multiple-answer items, one point was awarded if the participant accurately identified the scientifically recognized options. A cumulative score of ≥60% (corresponding to a score of ≥5 out of 7) was utilized as the cutoff to categorize participants as having "good knowledge." This threshold is justified and adapted based on the modified Bloom’s criteria commonly used in similar public awareness studies.

## Results

Table 1 presents the socio-demographic characteristics of 444 adult participants in Riyadh. The majority of respondents were aged 26-35 years (n=135, 30.4%), followed by 36-45 years (n=103, 23.2%) and 46-55 years (n=93, 20.9%), while 7.7% (n=34) were aged 56 years or older. Most participants were female (n=367, 82.7%), and the majority were married (n=287, 64.6%). Regarding education, more than half held a bachelor’s degree (n=258, 58.1%), while 22.1% (n=98) had secondary education or below. Considering employment, 41.2% (n=183) were working, 33.8% (n=150) were not working, and 25.0% (n=111) reported other work statuses. Nearly 38.7% (n=172) had a monthly income below 5,000 Saudi Riyals (SAR), while 32.0% (n=142) earned between 5,000-10,000 SAR, and 29.3% (n=130) reported more than 10,000 SAR. Concerning family composition, 38.1% (n=169) had no children, whereas 24.1% (n=107) had more than four. Finally, only 9.5% (n=42) of participants had a personal history of directly receiving formal clinical counseling about BHS from a healthcare provider (Table 1).

**Table 1:** Socio-Demographic Characteristics of Adult Participants in Riyadh (N=444) SAR: Saudi Riyals

Socio-Demographics	N	%
Age in years		
18-25	79	17.8
26-35	135	30.4
36-45	103	23.2
46-55	93	20.9
56 or more	34	7.7
Gender		
Male	77	17.3
Female	367	82.7
Marital status		
Single	127	28.6
Married	287	64.6
Divorced/widow	30	6.8
Educational level		
Secondary/below	98	22.1
Diploma	47	10.6
Bachelor degree	258	58.1
Post-graduate degree	41	9.2
Work status		
Not working	150	33.8
Working	183	41.2
Other	111	25.0
Monthly income		
<5000 SAR	172	38.7
5000-10000 SAR	142	32.0
>10000 SAR	130	29.3
Number of children		
No children	169	38.1
1-2 children	86	19.4
3-4 children	82	18.5
>4 children	107	24.1
Have you ever received information about breath-holding episodes from a healthcare provider?		
Yes	42	9.5
No	402	90.5

Less than half of respondents (n=197, 44.4%) had heard of BHS. Knowledge regarding affected age, causes, and symptoms was limited: 34.7% (n=154) correctly identified six months to two years as the peak age, crying was the most recognized trigger (n=336, 75.7%), and 79.5% (n=353) identified cyanosis or pallor as a key symptom. Awareness of the association with iron deficiency anemia was low (n=105, 23.6%). Overall, 75.7% (n=336) demonstrated poor knowledge, while 24.3% (n=108) had good knowledge based on the ≥60% cutoff score (Table 2).

**Table 2:** Knowledge and Awareness of Adults in Riyadh Regarding Breath-Holding Spells in Children (N=444) * Multiple responses were allowed for these items; percentages may exceed 100%.

Knowledge and Awareness Items	N	%
Have you ever heard of breath-holding spells in children?		
Yes	197	44.4
No	247	55.6
What age group is most affected by breath-holding spells?		
0-6 months	143	32.2
6 months-2 years	154	34.7
2-4 years	92	20.7
4-6 years	55	12.4
The main causes of breath-holding spells*		
Crying	336	75.7
Fear	210	47.3
Anger	169	38.1
Pain	101	22.7
Others	5	1.1
I don't know	4	0.9
Common symptoms of breath-holding spells*		
Being blue or pale	353	79.5
Loss of consciousness	185	41.7
Tremors or body stiffness	172	38.7
Tachypnea	108	24.3
I don't know	4	0.9
Breath-holding spells may be followed by epileptic seizures.		
Yes	88	19.8
No	55	12.4
I don't know	301	67.8
Risk factors associated with breath-holding spells*		
Genetic factors	140	31.5
Anemia	126	28.4
Emotional stress	215	48.4
Physical pain	222	50.0
I don't know	17	3.8
Breath-holding spells more common in children with iron deficiency anemia		
Yes	105	23.6
No	30	6.8
I don't know	309	69.6

**Figure 1:**
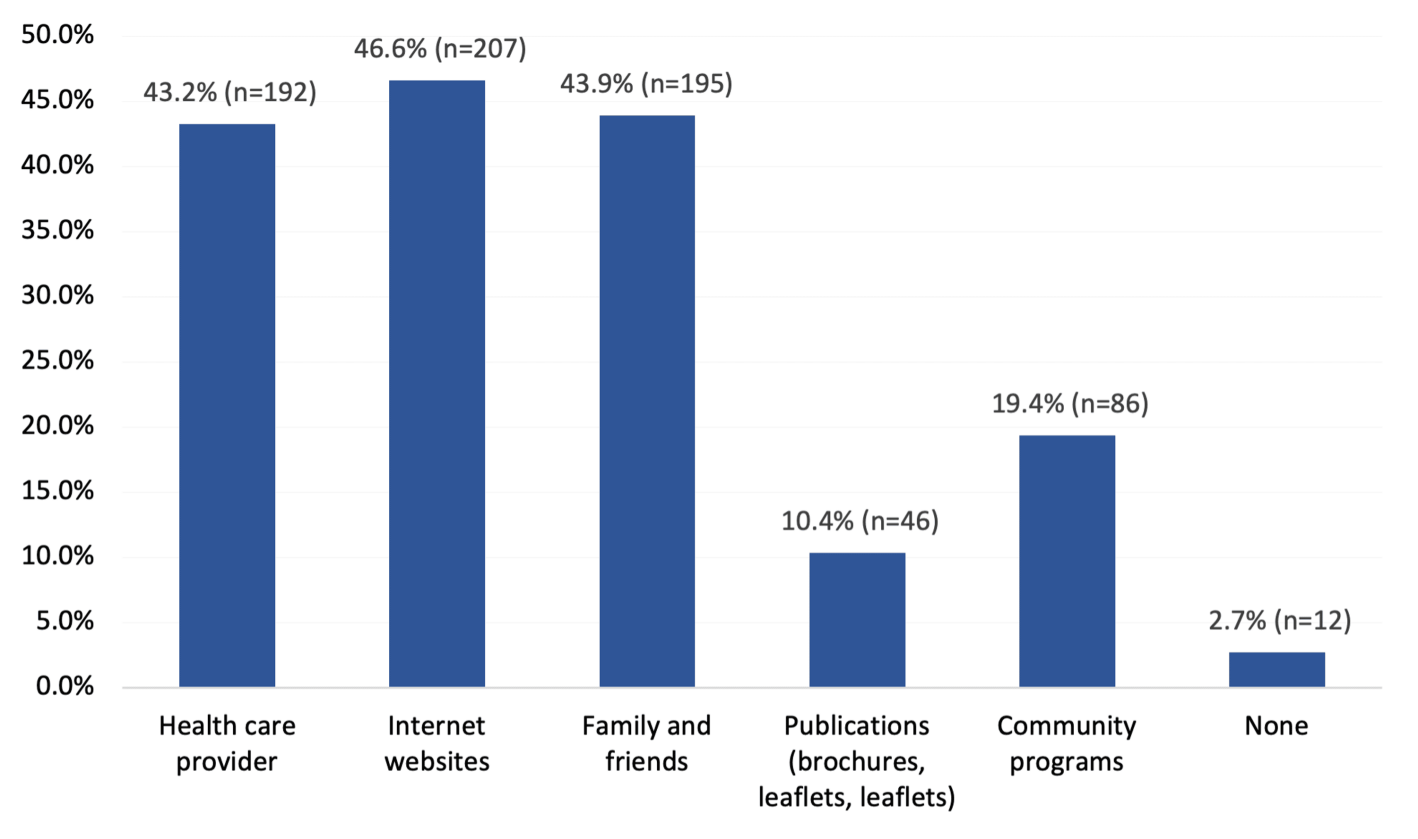
The Source of Participants' Information About Breath-Holding Spells in Children (N=444)

The main sources of information perceived as helpful were the internet (n=207, 46.6%), family/friends (n=195, 43.9%), and healthcare providers (n=192, 43.2%), although only 9.5% (n=42) had actually received direct BHS information from a provider previously (Figure 1).

Regarding attitudes, participants reported high concern: 76.4% (n=339) would experience severe anxiety if their child had apnea, and most were likely to seek medical advice for recurrent episodes (62.6%, n=278 very likely; 29.7%, n=132 likely) (Table 3).

**Table 3:** Attitudes of Adults in Riyadh Toward Breath-Holding Spells in Children (N=444)

Items	N	%
How worried would you be if your child had apnea?		
Not worried at all	7	1.6
Slight anxiety	23	5.2
Moderate anxiety	75	16.9
Severe anxiety	339	76.4
Do you think that a healthcare professional should always treat cases of breath-holding?		
Yes	207	46.6
Maybe	189	42.6
No	48	10.8
How likely is it that you should seek medical advice if your child has frequent breath-holding episodes?		
Very unlikely	17	3.8
Unlikely	17	3.8
Likely	132	29.7
Very likely	278	62.6

Age and marital status were the only socio-demographic factors significantly associated with knowledge (p<0.05). Younger adults (18-25 years) showed significantly higher knowledge, indicating a small-to-medium association (χ^2^=10.43, df=4, p=0.034, Cramer's V=0.15). Single participants were also more likely to have good knowledge, demonstrating a similar small-to-medium effect strength (χ^2^=9.71, df=2, p=0.008, Cramer's V=0.15). Conversely, negligible and non-significant associations were observed for gender (χ^2^=0.05, df=1, p=0.711, Cramer's V=0.01) and educational level (χ^2^=0.92, df=3, p=0.820, Cramer's V=0.04) (Table 4). The remaining demographic variables, including monthly income, number of children, and prior information from healthcare providers, were also statistically evaluated and demonstrated no significant association with the participants' knowledge level (p>0.05).

**Table 4:** Factors Associated With Adult Participants in Riyadh About Breath-Holding Spells in Children (N=444) P: Pearson χ^2^ test; ^: Exact Probability test; *: P<0.05 (significant); χ^2^: chi-square statistic; df: degrees of freedom

Factors	Poor (n=336)	Good (n=108)	χ^2^	df	Cramer's V	p-value
Age in years						
18-25	49 (62.0%)	30 (38.0%)	10.43	4	0.15	0.034*
26-35	104 (77.0%)	31 (23.0%)				
36-45	80 (77.7%)	23 (22.3%)				
46-55	75 (80.6%)	18 (19.4%)				
56 or more	28 (82.4%)	6 (17.6%)				
Marital status						
Single	84 (66.1%)	43 (33.9%)	9.71	2	0.15	0.008*^
Married	226 (78.7%)	61 (21.3%)				
Divorced/widow	26 (86.7%)	4 (13.3%)				
Gender						
Male	57 (74.0%)	20 (26.0%)	0.05	1	0.01	0.711
Female	279 (76.0%)	88 (24.0%)				
Educational level						
Secondary/below	74 (75.5%)	24 (24.5%)	0.92	3	0.04	0.820
Diploma	33 (70.2%)	14 (29.8%)				
Bachelor degree	198 (76.7%)	60 (23.3%)				
Post-graduate degree	31 (75.6%)	10 (24.4%)				
Work status						
Not working	116 (77.3%)	34 (22.7%)	0.63	2	0.04	0.728
Working	135 (73.8%)	48 (26.2%)				
Other	85 (76.6%)	26 (23.4%)				

Participants reported high levels of concern regarding BHS. Most would experience severe anxiety if their child had apnea, and the majority indicated they would likely seek medical advice for recurrent episodes (Table 5).

**Table 5:** Association Between Attitudes Toward BHS and Knowledge Level P: Pearson χ^2^ test; ^: Exact Probability test; χ^2^: Chi-square statistic; df: Degrees of freedom; BHS: breath-holding spells

Attitude Item	Poor (n=336)	Good (n=108)	χ^2^	df	Cramer's V	p-value
Worry about apnea						
Not worried at all	4 (1.2%)	3 (2.8%)	2.94	3	0.08	0.401^
Slight anxiety	20 (6.0%)	3 (2.8%)				
Moderate anxiety	56 (16.7%)	19 (17.6%)				
Severe anxiety	256 (76.2%)	83 (76.9%)				
Healthcare treatment						
Yes	153 (45.5%)	54 (50.0%)	1.88	2	0.07	0.391
Maybe	143 (42.6%)	46 (42.6%)				
No	40 (11.9%)	8 (7.4%)				
Likelihood of seeking advice						
Very unlikely	13 (3.9%)	4 (3.7%)	2.95	3	0.08	0.399^
Unlikely	15 (4.5%)	2 (1.9%)				
Likely	104 (31.0%)	28 (25.9%)				
Very likely	204 (60.7%)	74 (68.5%)				

The findings indicate no statistically significant differences in attitudes between participants with good and poor knowledge, highlighting uniformly high concern across all knowledge levels (p>0.05).

## Discussion

The present study revealed that less than half of the participants (n=197, 44.4%) had ever heard of BHS. This finding reflects a considerable deficit in public awareness in Riyadh, despite BHS being one of the most common paroxysmal events in early childhood [1]. Comparable studies in other regions have reported similarly low levels of awareness among parents. A study in Saudi Arabia found that only 39% of mothers were familiar with BHS, with most relying on informal sources of information rather than healthcare providers [13]. In contrast, research from Turkey revealed slightly higher awareness levels, where nearly 55% of parents had prior knowledge of BHS, reflecting differences in health education and pediatric counseling practices [14]. These variations indicate the importance of culturally tailored awareness campaigns.

In our study, the majority of respondents correctly identified six months to two years as the most affected age group (n=154, 34.7%). This matches with the established epidemiology of BHS, which typically manifests between 6 and 48 months, peaking around the second year of life [1,2]. However, a considerable proportion incorrectly selected the 0-6-month age group, with confusion about the early onset of the condition. Previous literature highlights that while rare neonatal cases have been reported [11], the vast majority occur after six months of age. Misconceptions about the age of onset may contribute to unnecessary parental anxiety when infants younger than six months present with unrelated apnea or cyanotic episodes.

Crying was the most frequently recognized cause (n=336, 75.7%), followed by fear and anger. These findings are consistent with the literature, which identifies emotional triggers such as frustration, anger, and fear as the most common precipitants of cyanotic BHS [2,15]. However, nearly one-quarter of participants recognized pain as a potential trigger (n=101, 22.7%), despite evidence that minor trauma and painful stimuli are well-documented precipitants of pallid BHS [16,4]. This under-recognition may reflect the predominance of cyanotic over pallid spells in clinical practice, leading to skewed public perceptions.

The most commonly identified symptom was being blue or pale (n=353, 79.5%), which is in line with the defining clinical features of cyanotic and pallid BHS [15,17,18,4]. Awareness of more severe manifestations was lower: less than half recognized loss of consciousness (n=185, 41.7%), and about one-third identified tremors or stiffness (n=172, 38.7%). These defects are important, as convulsive movements and transient loss of consciousness are often misinterpreted as epileptic seizures [19]. Similar misconceptions have been reported in studies from Egypt and Jordan, where parents frequently confused BHS with epilepsy, leading to unnecessary investigations and treatments [20-22]. Improving public understanding of the benign and self-limiting nature of these symptoms is therefore essential.

Only one-fifth of participants (n=88, 19.8%) believed BHS may be followed by epileptic seizures, while the majority reported not knowing. This uncertainty reflects the ongoing challenge of differentiating BHS from epilepsy in both public and clinical contexts. Although seizure-like activity may occur during severe spells due to cerebral hypoxia, true epilepsy is rare in children with BHS [2,5]. Studies from other regions indicated that misdiagnosis of epilepsy in children with BHS remains common, often resulting in unnecessary antiepileptic therapy [8,9]. Our findings mean that public education should know the distinction between BHS and epilepsy to reduce stigma and inappropriate management.

Physical pain and emotional stress were the most frequently reported risk factors, while fewer participants recognized genetic predisposition or anemia. This is concerning, as iron deficiency anemia is one of the most consistently reported risk factors for BHS, with supplementation shown to reduce frequency and severity of spells [23,24]. In our study, only one-fourth (n=105, 23.6%) were aware of this association, indicating a critical knowledge gap.

Overall, three-quarters of participants (n=336, 75.7%) showed poor knowledge, with only 24.3% (n=108) achieving good awareness. This reflects findings from other Middle Eastern studies, where parental knowledge of BHS was generally low [12,13,25]. The reliance on non-medical sources of information, such as the internet (n=207, 46.6%) and family/friends (n=195, 43.9%), further underscores the limited role of healthcare providers in providing accurate information. In our study, only 9.5% of participants (n=42) had ever received information about BHS from a healthcare professional. This represents a missed opportunity for preventive education during routine child health visits.

Considering factors associated with participants' knowledge, our study revealed that age and marital status were the only socio-demographic factors significantly associated with knowledge of BHS. Younger adults (18-25 years) showed the highest level of good knowledge (n=30, 38.0%), with awareness decreasing as age increased. Similar findings have been reported in other studies, where younger individuals showed better awareness due to greater exposure to online health resources [26,27]. Likewise, single participants exhibited higher knowledge (n=43, 33.9%) compared to married (n=61, 21.3%) and divorced/widowed (n=4, 13.3%) individuals. This trend could be explained by the fact that single individuals in our study were primarily clustered within the younger age bracket, a demographic that actively utilizes digital health resources, social media infographics, and online forums where medical awareness is frequently shared. Furthermore, before transitioning into parenthood, single individuals may possess more discretionary time to engage with diverse educational materials online, whereas married parents are often consumed by the immediate, time-intensive demands of active childcare, leaving less opportunity for elective health reading. Other variables, including gender, education, work status, income, number of children, and previous information from healthcare providers, were not significantly related to knowledge. However, it is highly probable that the observed association with marital status is confounded by age, as individuals in the younger age bracket (18-25 years) are more likely to be single and inherently more active in seeking digital health information.

Also, the present study revealed that most participants experienced severe anxiety when their child had an episode of apnea (n=339, 76.4%), reflecting considerable emotional distress. This reaction is consistent with an assessed low awareness level. Many respondents believed that a healthcare professional should always treat BHS, while others remained uncertain, indicating limited confidence in distinguishing benign spells from more serious conditions such as epilepsy or syncope. Participants also expressed a strong tendency to seek medical advice if their child experienced frequent spells (n=278, 62.6% very likely; n=132, 29.7% likely). While this proactive approach ensures proper evaluation for underlying causes like iron deficiency anemia, it may also lead to unnecessary healthcare visits when awareness of the harmless course of BHS is limited. Importantly, no significant association was found between attitudes and overall knowledge, suggesting that emotional responses to BHS are largely shaped by the dramatic presentation rather than factual understanding.

This study has several limitations that should be acknowledged. First, the cross-sectional design limits our ability to establish causal relationships between socio-demographic factors and knowledge levels. Second, the use of an online self-administered questionnaire may introduce selection bias, as it excludes individuals without internet access or those less digitally literate. Third, the study sample exhibited a significant gender imbalance, with a high proportion of female respondents (82.7%). While this skew is common in pediatric and childcare-related surveys due to the traditionally higher engagement of mothers and female caregivers in child health issues, it may limit the generalizability of the findings to the male population. Fourth, the assessment of parental anxiety was based on a subjective, self-reported scale rather than an objective, clinically validated anxiety instrument, which may introduce individual variance in interpreting severity levels. Fifth, the statistical analysis was restricted to bivariate tests (chi-square), which evaluate unadjusted associations and cannot control for potential confounding factors, such as the close interplay between younger age and single marital status. Sixth, the data are based on self-reports, which may be subject to recall bias. Finally, since the study was conducted only in Riyadh, the findings may not be fully generalizable to the entire population of Saudi Arabia, particularly those in rural areas.

Despite these limitations, this study possesses several notable strengths. First, it utilizes an adequate and statistically robust sample size (N=444), which enhances the reliability of the observed trends. Second, it focuses on BHS, a clinically significant yet relatively under-studied pediatric topic in Saudi Arabia, thereby filling an important gap in the local public health literature. Finally, rather than limiting the scope to basic awareness, this study provides a comprehensive assessment by simultaneously evaluating both the knowledge levels and the specific practical attitudes of participants, offering a more holistic view of parental readiness to manage this frightening condition.

## Conclusions

In conclusion, the study revealed that knowledge and awareness regarding BHS among adults in Riyadh remain generally low, with only a minority having an adequate understanding of the condition. While most participants recognized crying, fear, and anger as common triggers and identified cyanosis or pallor as key symptoms, awareness of underlying risk factors was clearly insufficient. The internet and social networks were the main sources of information, reflecting limited contact with healthcare professionals or structured educational programs. Attitudinally, participants showed a high level of concern and anxiety toward BHS episodes, confirming the emotional burden experienced by caregivers. Public health strategies should aim to raise awareness about the benign nature of BHS and their link to iron deficiency anemia. Healthcare providers should actively educate parents during routine visits, offering reassurance and guidance on management. Educational campaigns should use online platforms and community programs to reach a wider audience, especially younger caregivers.

## Appendices

Study questionnaire

Section 1: Socio-Demographic Characteristics

1. Age in years: (18-25, 26-35, 36-45, 46-55, 56 or more)

2. Gender: (Male, Female)

3. Marital status: (Single, Married, Divorced/widow)

4. Educational level: (Secondary/below, Diploma, Bachelor's degree, Post-graduate degree)

5. Work status: (Working, Not working, Other)

6. Monthly income: (< 5000 SR, 5000-10000 SR, > 10000 SR)

7. Number of children: (No children, 1-2 children, 3-4 children, > 4 children)

8. Have you ever received information about breath-holding episodes from a healthcare provider? (Yes, No) *Section 2: Knowledge and Awareness*

9. Have you ever heard of breath-holding spells in children? (Yes, No)

10. What age group is most affected by breath-holding spells? (0-6 months, 6 months-2 years, 2-4 years, 4-6 years)

11. The main causes of breath-holding spells: (Crying, Fear, Anger, Pain, Other)

12. Common symptoms of breath-holding spells: (Being blue or pale, Loss of consciousness, Tremors or body stiffness, Tachypnea, Other)

13. Breath-holding spells may be followed by epileptic seizures? (Yes, No, I don't know)

14. Risk factors associated with breath-holding spells: (Genetic factors, Anemia, Emotional stress, Physical pain, Other)

15. Breath-holding spells more common in children with iron deficiency anemia: (Yes, No, I don't know) *Section 3: Attitudes*

16. How worried would you be if your child had apnea? (Not worried at all, Slight anxiety, Moderate anxiety, Severe anxiety)

17. Do you think that cases of breath-holding should always be treated by a healthcare professional? (Yes, Maybe, No)

18. How likely is it that you should seek medical advice if your child has frequent breath-holding episodes? (Very unlikely, Unlikely, Likely, Very likely)

19. Sources of information have you found most helpful in learning about breath-holding spells: (Health care provider, Internet websites, Family and friends, Publications, Community programs, Other)
